# Impact of Dietary Variations on Kuruma Shrimp (*Penaeus japonicus*) Assessed through Individual-Based Rearing and Insights into Individual Differences

**DOI:** 10.3390/ani14152267

**Published:** 2024-08-04

**Authors:** Chuanxi Chen, Chunxiang Ai, Wenzhi Cheng, Huiyang Huang, Yiling Hou, Xiaojie Deng, Siqi Li, Yue Liu, Peng Xu, Yong Mao

**Affiliations:** 1 State Key Laboratory of Marine Environmental Science, College of Ocean and Earth Sciences, Xiamen University, Xiamen 361102, China; chuanxichen@stu.xmu.edu.cn (C.C.); chunxai@xmu.edu.cn (C.A.); huiyang@xmu.edu.cn (H.H.); 22320211151348@stu.xmu.edu.cn (Y.H.); dengxiaojiekl@126.com (X.D.); 22320221151402@stu.xmu.edu.cn (S.L.); ly590327@163.com (Y.L.); 2 State Key Laboratory of Mariculture Breeding, College of Ocean and Earth Sciences, Xiamen University, Xiamen 361102, China; 3 Fujian Key Laboratory of Genetics and Breeding of Marine Organisms, College of Ocean and Earth Sciences, Xiamen University, Xiamen 361102, China; 4 Department of Computer Science, Xiamen University, Xiamen 361102, China; chengwenzhi@xmu.edu.cn; 5 National Observation and Research Station for the Taiwan Strait Marine Ecosystem, Zhangzhou 363400, China

**Keywords:** *Penaeus japonicus*, live feed, formulated feed, growth performance, individual-rearing, intestine microbiota

## Abstract

**Simple Summary:**

The individual-rearing method has proven beneficial for detailed studies on the growth and feeding of aquatic organisms, helping to minimize the impact of non-dietary factors such as cannibalism. The Kuruma shrimp *Penaeus japonicus* occupies a significant niche in global aquaculture, but there is still a gap in the research on its nutrient supply, particularly in comparing the nutritional impacts of live feed and pellet diets. This study employed an individual-rearing method to investigate the effects of live feed (*Perinereis aibuhitensis*), formulated pellet diets, and their combinations on the growth and health of *P. japonicus*. The results revealed that live feed can enhance shrimp growth performance compared to pellet diets and provide a healthier and more stable intestinal flora. Growth and feeding performance on a mixed diet were comparable to live feed while also reducing costs. And the individual-rearing method allowed us to find inter-individual differences in growth and feeding, with daily intake varying cyclically with the molting cycle. These findings elucidate the feed preferences as well as the growth and feeding characteristics of shrimp, offering novel methodologies for shrimp feed selection, trait collection, and breeding strategies. This has the potential to significantly enhance aquacultural practices.

**Abstract:**

This study developed an individual-rearing method to compare the effects of live feed (sandworms *Perinereis aibuhitensis*), formulated pellet diets, and a mixture of live feed and formula feed on the Kuruma shrimp *Penaeus japonicus*, aiming to minimize the influence of non-dietary factors on the growth of *P. japonicus*, like cannibalism. Results indicated that live feed, with its higher protein, essential amino acids, and fatty acid content, led to significantly better growth and feeding performance in *P. japonicus* (*p* < 0.05) compared to pellet diets. A mixed diet resulted in a lower average daily protein intake yet maintained a growth and feeding performance comparable to live feed. The intestinal microbiota of shrimp, dominated by Proteobacteria, Bacteroidetes, Firmicutes, and Actinobacteria, showed significant shifts with diet changes. Specifically, formulated feed increased the relative abundance of *Vibrio* and *Photobacterium* while decreasing *Shimia* and Rhodobacterales (*p* < 0.05), and feeding live food resulted in a more complex and stable bacterial network. Notably, individual variances in growth and feeding were observed among shrimps, with some on formulated diets showing growth comparable to those on live feed. Each shrimp’s final weight, specific growth rate, protein efficiency rate, and average daily food intake positively correlated with its initial body weight (*p* < 0.05), and daily intake varied cyclically with the molting cycle. These findings suggest that individual-rearing is an effective approach for detailed feed evaluation and monitoring in *P. japonicus*, contributing to improved feed selection, development, and feeding strategies.

## 1. Introduction

Shrimps have historically been among the most widely traded aquatic commodities, constituting 17% of the global value of all aquatic product exports in 2022, playing a crucial role in the aquaculture sector of many countries [1,2,3]. The *Kuruma shrimp*, *Penaeus japonicus*, is a key species in both trade and aquaculture, extensively cultivated in the Indo-West Pacific region, including China, Australia, the Philippines, and Japan, due to its high commercial value and palatable flavor [4,5,6,7]. With increasing market demand and a decline in wild catch, aquaculture has become pivotal in meeting the demand for this high-valued seafood [8,9].

The nutritional aspects comprise one of the most critical components in aquaculture production, as these influence biological performance and enterprise profitability [10]. In the early stages of *P. japonicus* cultivation, commonly used live foods include frozen squids, oysters, clams, mussels, and polychaetes. These feeds are rich in EPA, DHA, and protein, making them easily digestible and absorbed by the shrimp, thereby promoting their growth [11,12,13]. However, scaling up cultivation has raised challenges with live feed supply and biosecurity, limiting industry growth [14]. Consequently, to enable a scientific, high-quality, and sustainable shrimp culture, researchers employed refined test diets to reveal the nutritional requirements of *P. japonicus* for adequate proteins, lipids, carbohydrates, minerals, and vitamins, like other aquatics [15,16]. Building upon previous research on the nutritional requirements of *P. japonicus*, researchers have extensively investigated the effects of varying nutrient compositions [17,18,19] and nutrient sources [8,9,20,21] on the growth, immunity, and digestion of *P. japonicus*. These studies have contributed to the formulation of numerous compounded diets for *P. japonicus*, thereby supporting the rapid expansion of the Kuruma shrimp culture. Nevertheless, some studies have found that shrimp-fed formulated feeds grow more slowly than those fed live feeds during the juvenile stage of the species [22,23]. However, comparative studies on live feed and formulated feed for shrimp are still limited.

A vast number of analytical techniques and monitoring methods have shed light on the nutritional performance of experimental diets and feeding regimes designed for aquatic organisms at different life stages growing under a variety of production systems [10]. As shrimp are generally reared in large groups in water, macro and micro methods have been used to study their growth, nutrition, and molting under group-rearing conditions [24,25,26,27]. However, some results, especially for metrics such as feed-influenced survival rates, can be inaccurate due to uncontrolled intraspecific interactions, including cannibalism of the shrimp [28]. Aquatic organism studies have employed individual-rearing methods to reduce the impact of confounding factors like group competition and enable the accurate monitoring of each individual’s growth and feeding performance, thus improving the accuracy of experimental results [29,30,31,32]. In shrimp, researchers have employed individual-rearing methods to collect growth and feeding information of individual shrimp, which has been used for the precise evaluation of feed efficiency traits. This approach has been successfully applied in breeding programs for *Litopenaeus vannamei* and *Fenneropenaeus chinensis* [27,31,33]. However, there is a lack of research evaluating shrimp feed using individual-rearing methods, particularly concerning the growth and feed of *P. japonicus*.

This study employed the individual-rearing method to compare the effects of live feed (sandworms *Perinereis aibuhitensis*) and formulated pellet diets on development and feeding performance of *P. japonicus* and analyze their individual variation. A feeding trail with three different feeding regimes was conducted with shrimp fed either sandworms exclusively, a formulated pellet diet only, or a mix of a sandworm-formulated diet. The objectives were to (1) use the individual-rearing method to assess the effects of sandworms, formulated diet, and mixed feeding on *P. japonicus* growth, digestion, immunity, intestine microbiota, and feeding behavior; (2) Use the individual-rearing method to collect more precise data on the growth and feeding of *P. japonicus* and to confirm that the strategy can be used to evaluate the growth and feed of this species.

## 2. Materials and Methods

### 2.1. Animals Acquisition

All *P. japonicus* shrimps were obtained from a full-sib family in an aquaculture farm in Dongshan (Zhangzhou, China) in November 2022 and acclimated in environmentally controlled small tanks with a recirculating water system with aerated seawater at 26 °C and salinity of 31‰ prior to experimentation in the National Observation and Research Station for the Taiwan Strait Marine Ecosystem, Dongshan Swire Marine Station (Zhangzhou, China). The in situ seawater supply system provided filtered, flowthrough seawater, and shrimps were fed the right amount of bait daily at 17:00.

### 2.2. Culture System and Experimental Design

**Figure 1:**
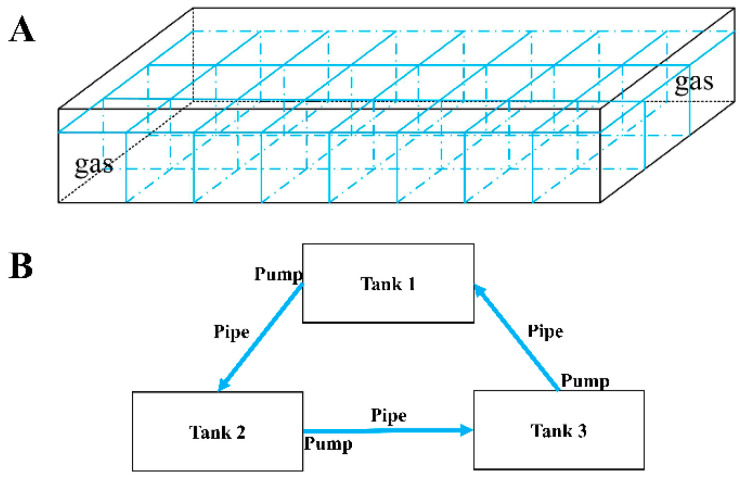
Individual-rearing device (**A**). Tank connection and distribution pattern during the experiment (**B**).

The individual-rearing system was used during the experiment. There were 3 independent tanks (116 cm × 36 cm × 15 cm) with a water volume of about 62 L per tank. Twenty-four separate spaces (145 mm × 120 mm × 150 mm) were created in each tank using plastic plates with holes (Figure 1A). The three tanks had a common experimental environment, except for their daily diets, since pipes connected them in series and used pumps to support an adequate exchange in water flow to create a stable internal circulation system (Figure 1B). The temperature was maintained at 26.0 °C ± 0.5 °C, and seawater was exchanged by 80% daily.

Prior to the experiment, each tank was randomly loaded with 20 healthy shrimp (2.16 ± 0.18 g in body weight; the remaining 4 separate spaces were used to place heating rods and air stones). After being placed in the tanks, all shrimps had a one-week adaptation to the experimental environment. The experiment then proceeded for 4 weeks. Dead shrimp and molts were promptly removed. Three diet groups—the first, a diet of 1.2 mm diameter formulated pellet diets (group F) purchased from Fujian Haida Feed Co., Ltd. (Zhangzhou, China), known for its high protein content, widely used by local farmers, and can remain stable in pellet form for at least one day; the second, live food (*P. aibuhitensis*, group N) purchased from an aquaculture farm in Dongshan (Zhangzhou, Fujian); the third, a 1:1 mixture of pellet diets and live food (by wet weight, group NF)—were created during the experiment. These diets were hand-fed to different tanks at 17:30 each day according to these shrimps’ inactive habits. Taking each tank as a unit, feed for each individual was stored independently. During the experiment, an appropriate feed dose per meal was ensured for their apparent satiation, and the amount of daily feed intake of the shrimp was equal to the weight difference between the feed supply and the remaining diets. The remaining bio-diets were also recorded in the same way. Based on previous breeding and experimental experiences, the live food was all fresh *P. aiibuhitensis*, which was disinfected with iodophor to eliminate potential pathogens and washed with clean water before feeding. The remaining pellet diets were thoroughly dried and weighed. The number of shrimps in each group was counted at the end of the experiment to determine each group’s survival rate.

Following 24 h of starvation after the feeding trial, the animals were weighed individually. Subsequently, 15 shrimp were then randomly taken from each tank, and the entire body surface was disinfected using some drenched cotton dipped in 75% ethanol. The shrimps were then dissected on ice to collect the hepatopancreas, intestine, and muscle samples, and all of these samples were stored at −80 °C for analysis of the enzyme and immunity activities. In addition, the pellet diets and live foods were collected to analyze the nutrient composition.

### 2.3. Growth Performance

Body weight (BW) was recorded for all animals at the start (IBW) and end (FBW) of the experiment, respectively. During the experiment, the molting time was recorded to calculate the molt cycle of every animal. The daily feed intake (DFI) and feed intake (FI) of each animal were also recorded. The body weight gain (BWG), body weight gain rate (WGR), specific growth rate (SGR), feed efficiency ratio (FER), and protein efficiency rate (PER) were calculated as follows:

(1) BWG (g)=FB (g) − IB (g).

(2) WGR (%)=100 × [FB (g)−IB (g)]/IB (g).

(3) FER=BWG (g)/FI (g).

(4) PER=BWG (g)/protein intake (g).

(5) SGR=[ln FB (g)−ln IB (g)]/days × 100.

### 2.4. Analysis of Enzyme Activity and Antioxidant Capacity

The hepatopancreas was used to analyze the immunological, digestive, and antioxidant enzymes. The total antioxidant capacity (T-AOC and colorimetry), total superoxide dismutase (T-SOD and hydroxylamine method), malondialdehyde (MDA and thiobarbituric acid (TBA)), catalase (CAT and ammonium molybdate method), glutathione Peroxidase (GSH-Px and colorimetric method), reduced glutathione (GSH and microplate method), alkaline phosphatase (AKP and visible light colorimetry), phenoloxidase (PO and competition method), lysozyme (LZM and turbidimetry), lipase (LPS and colorimetry), Trypsin (colorimetry), and α-amylase (AMS and starch-iodine colorimetry) were measured using respective kits (Nanjing Jiancheng Bioengineering Institute, Nanjing, China) according to the manufacturer’s instructions.

### 2.5. Dietary Proximate Composition Analysis

The proximate composition of the diets was evaluated according to the standard procedure published in AOAC [34]. In brief, moisture was determined with the drying method at 105 °C, crude protein (nitrogen × 6.25) was analyzed by the Kjeldahl method after acid digestion, and crude lipid was determined by Soxhlet extraction.

### 2.6. Dietary Amino Acid and Fatty Acids Composition

Freeze-dried diet samples (approximately 100 mg) were put into tubes with 25 mL trichloroacetic acid (5 g/100 mL) and then kept at 4 °C for 2 h. Approximately 0.4 mL of the supernatant was collected after centrifugation (15,000 rmp, 30 min, 4 °C) and filtered through a 0.22 μm membrane, and then analyzed for free amino acid composition using an L-8900 amino acid analyzer (Hitachi, Japan).

The diet samples (approximately 200 mg) were added to a 10 mL glass tube with 3 mL chloroform/methanol (2:1 by volume). The extracted fats were mixed with 2 mL KOH-methanol (c = 0.5 mol/L) and reacted in 50 °C water for 10 min. After cooling for 3 min, 2 mL of BF3-methanol solution (w = 10%) was added to the mixture and incubated in a water bath at 80 °C for 20 min. Then, 1 mL n-hexane and 2 mL saturated NaCl solution were added to the above mixture. The solution was shaken vigorously to promote layer separation and centrifuged at 1500 rpm for 5 min, and the supernatant was filtered through a 0.22 μm ultrafiltration membrane and collected in a 1.5 mL ampoule bottle. Finally, the obtained fatty acid methyl esters were analyzed using a GC2010 plus gas chromatograph (Shimadzu, Japan). Methyl tridecanoate (Sigma, St. Louis, MO, USA) served as the internal standard. The results were presented as the relative percentages of each fatty acid (% total fatty acids).

### 2.7. Intestinal Microbial Analysis

Total bacterial DNA from all shrimp intestine samples was extracted by the CTAB method. The V4–V5 region of 16S rRNA genes was amplified by PCR using primers 515F (5′GTGCCAGCMGCCGCGGTAA 3′) and 806R (5′CCGTCAATTCCTTTGAGTTT 3′). Sequencing libraries were generated using a TruSeq^®^ DNA PCR-Free Sample Preparation Kit (Illumina, Inc., San Diego, USA). The library was checked with Qubit, and a real-time PCR was used for quantification. A bioanalyzer was also used for size distribution detection. Quantified libraries were pooled and sequenced on Illumina platforms according to an effective library concentration and the amount of data required. The sequences obtained in this study are available in the NCBI SRA database with the accession number PRJNA1059777.

Quality filtering of the raw tags was performed using the fastp (Version0.23.1) software to obtain high-quality clean tags [35]. The tags were compared with the reference database (Silva database (16S/18S), https://www.arb-silva.de/ (accessed on 24 February 2023); Unite Database (ITS), https://unite.ut.ee/ (accessed on 24 February 2023)) using UCHIME Algorithm (http://www.drive5.com/usearch/manual/uchime_algo.html (accessed on 24 February 2023)) to detect chimera sequences, and then the chimera sequences were moved and the effective tags were finally obtained [36]. For the obtained Effective Tags, denoise was performed with the DADA2 module in the QIIME2 software (Version QIIME2-202006) to obtain initial ASVs (Amplicon Sequence Variants), and then ASVs with an abundance of less than 5 were filtered out [37]. Species annotation was performed using the QIIME2 software based on the silva database (https://www.arb-silva.de/ (accessed on 24 February 2023)), and the taxa relative abundances of community compositions in samples were identified at different levels (phylum, class, order, family, and genus), respectively, and displayed with R software (version 4.2.3).

Alpha diversity indices, including Chao1, Shannon indexes, Simpson, and Dominance, were calculated by the QIIME2 software. The beta diversity among bacterial communities was evaluated using un-weighted Unifrac distances and visualized via non-metric multi-dimensional scaling (nMDS), which was plotted in R software. In addition, we screened ASVs with a relative abundance greater than 0.1% and calculated microbial co-occurrence network metrics using the *WGCNA* package, and the co-occurrence networks were assessed using the R package *igraph* 1.2.6 and visualized using *Gephi* 0.10.

### 2.8. Statistical Analysis

All of the statistical analyses were performed using SPSS software (version 21.0). After verifying normality and homogeneity of variances using Levene’s test, one-way ANOVA was used to evaluate the effects of different diets. Differences between treatments were compared using Tukey’s test when the effect was significant (*p* < 0.05). Differences are denoted as significant at *p* < 0.05, very significant at *p* < 0.01, and extremely significant at *p* < 0.001. Levene’s test for equality of variances was performed to ensure homogeneity of variances. The relationships between the final weight, specific growth rate, protein efficient rate, and average daily food intake versus the initial weight in *P. japonicus* and the relationship between the feed conversion ratio and daily feed intake versus individuals’ specific growth rate were calculated using Pearson’s correlation analysis. Results are shown as the mean ± standard deviation (S.D.).

## 3. Results

### 3.1. Nutrient Composition of the Diets

The proximate analysis shows that the protein content in sandworms was 12.27% higher than that in the formulated pellet diet (59% and 46.73%, respectively), and the lipid content followed a similar trend (10.73% and 8.90%, respectively, Table 1). The fatty and amino acid profiles are also presented in Table 1. Among the amino acids, the essential amino acid content of sandworms was higher than that of the pellet diet (18.86% and 16.22%, respectively). Among the fatty acids, sandworms had a higher content of highly unsaturated fatty acids compared to the pellet diet (1.13% and 0.42%, respectively).

### 3.2. Shrimp Feeding and Growth Performance

**Figure 2:**
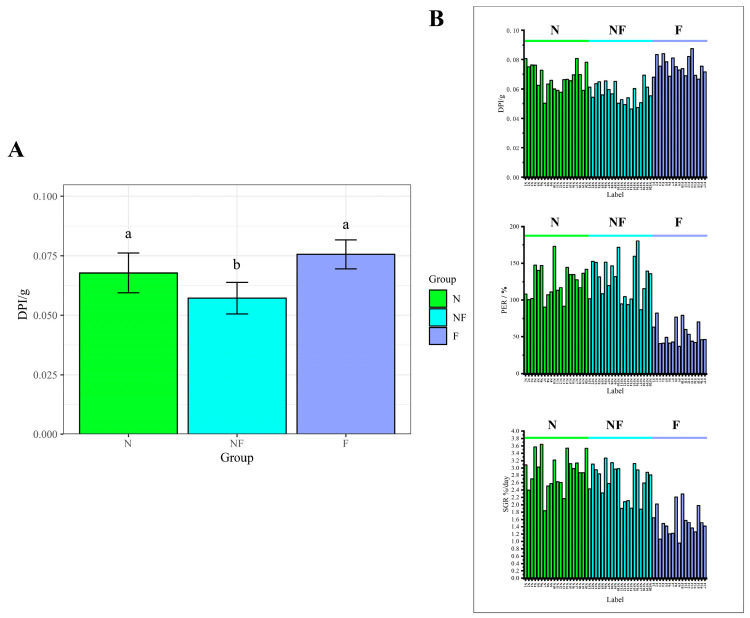
Comparison of daily protein intake (DPI) between three groups (**A**). Histogram of DPI, PER, and SGR for different individuals in different groups (**B**). (Different colors represent different groupings, which are N, NF, and F; data with different letters indicated significant differences (*p* < 0.05)).

After a four-week feeding trial, the growth performance of shrimps on different diets varied. Shrimps in groups N and NF had final body weights significantly higher than the shrimp in group F (*p* < 0.001) at the end of the experiment. Various diets significantly influenced the WG and SGR; the findings revealed that the WG and SGR of shrimps in groups N and NF did not differ significantly (*p* > 0.05) but were significantly greater than shrimps in group F (*p* < 0.001). While the PER of shrimp in groups N and NF did not differ significantly from one another, both were significantly higher than in group F. The FER of shrimp in group NF was significantly higher than that of the other two groups. Notably, group NF shrimps showed the lowest daily protein intake (DPI) in comparison to the other two groups (*p* < 0.05, Figure 2A). Additionally, during the experimental period, three shrimp in group F died as a result of an unsuccessful attempt to molt (survival rate: 85%, Table 2); one of them died halfway through the molt on day 13th of the experiment, and the other two died after the molt with soft bodies and without forming a new exoskeleton on day 20th of the experiment. Still, the shrimp in the other two groups remained alive. The results show that all of the shrimp in groups N and NF underwent three moltings, whereas only 57.89% of the shrimp in group F underwent three moltings (*p* < 0.05).

**Figure 3:**
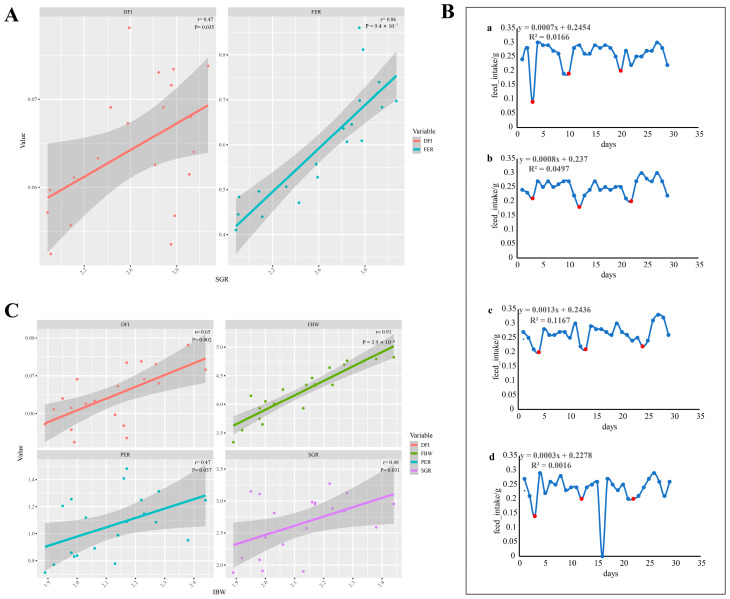
Pearson’s correlations of variation coefficients of feed efficiency ratio (FER) and daily feed intake (DFI) for shrimp individuals’ specific growth rate (SGR) (**A**). Changes in daily feed intake during the experiment (**B**). Relationship between initial body weight (IBW) and average daily food intake (DFI), final body weight (FBW), protein efficient rate (PER), and specific growth rate (SGR), and of the experiment shrimp housed individually (**C**). (a, b, c, d represent 4 shrimp from group NF respectively, and the red dots represent shrimp molting on this day.).

Furthermore, the growth and feeding behaviors of the shrimp also showed a significant level of inter-individual variation; notably, while the overall PER and SGR of the F group were lower than that of the N and NF groups, some individuals within the F group exhibited a PER and SGR comparable to the average levels observed in the NF and N groups (Figure 2B). According to every individual’s growth and feeding performance, a strong correlation was found between FER and SGR (*p* < 0.001, *r* = 0.86, Figure 3A), and a similar correlation was found between the DFI and SGR (*p* < 0.05, *r* = 0.47, Figure 3A). The results show that the DFI had a cyclical trend throughout the entire molting cycle. Although feeding activities persisted, the DFI of the shrimp peaked on the molting day of each cycle. The overall daily feeding of the shrimp showed an increasing tendency during the cultural experiment (Figure 3B). It is also noteworthy that the shrimp’s average daily food intake, final body weight, protein efficiency ratio, and specific growth rate showed positive linear correlations with its initial body weight (Figure 3C, with correlation coefficients of *p* < 0.01, *r* = 0.65; *p* < 0.001, *r* = 0.91; *p* < 0.05, *r* = 0.47; and *p* < 0.05, *r* = 0.48).

### 3.3. Activities of Digestive Enzymes

The activities of ASM and lipase were significantly higher in group N and group NF compared to group F (Table 3, *p* < 0.05). Additionally, the activities of Trypsin in group N exhibited the highest levels, although no significant difference was observed between group NF and group F (Table 3, *p* > 0.05).

### 3.4. Activities of Immunity Enzyme and Antioxidant Capacity

The activities of AKP in group N and group NF were significantly elevated compared with group F (Table 4, *p* < 0.01). Similarly, the activity of PO in group N and group NF was significantly higher than in group F (Table 4, *p* < 0.05). There were no significant differences in LZM levels among all groups (Table 4, *p* > 0.05). The highest T-SOD was observed in group N, while group NF and group F exhibited significantly reduced T-SOD levels (Table 4, *p* < 0.001). Furthermore, the activities of CAT in group N were the highest, whereas group F exhibited the lowest levels (Table 4, *p* < 0.05). The activity of GSH-Px was highest in group N, and the lowest activity was observed in group F (Table 4, *p* < 0.05). The GSH levels of shrimp in group N and group NF were significantly higher than those in group F (Table 4, *p* < 0.01). No significant difference in MDA levels was observed among the three groups (Table 4, *p* > 0.05).

### 3.5. Diversity, Taxonomic Composition, and Co-Occurrence Network Analysis of Intestinal Microbiota

A total of 750,405 high-quality, effective sequencing reads were obtained from 12 samples of *P. japonicus* guts, ranging from 36,169 to 73,050, and the average length of the sequences was 345 bp. A total of 887 amplicon sequence variants (ASVs) were obtained, and the number of ASVs increased from 38 to 220 (Table 5).

**Figure 4:**
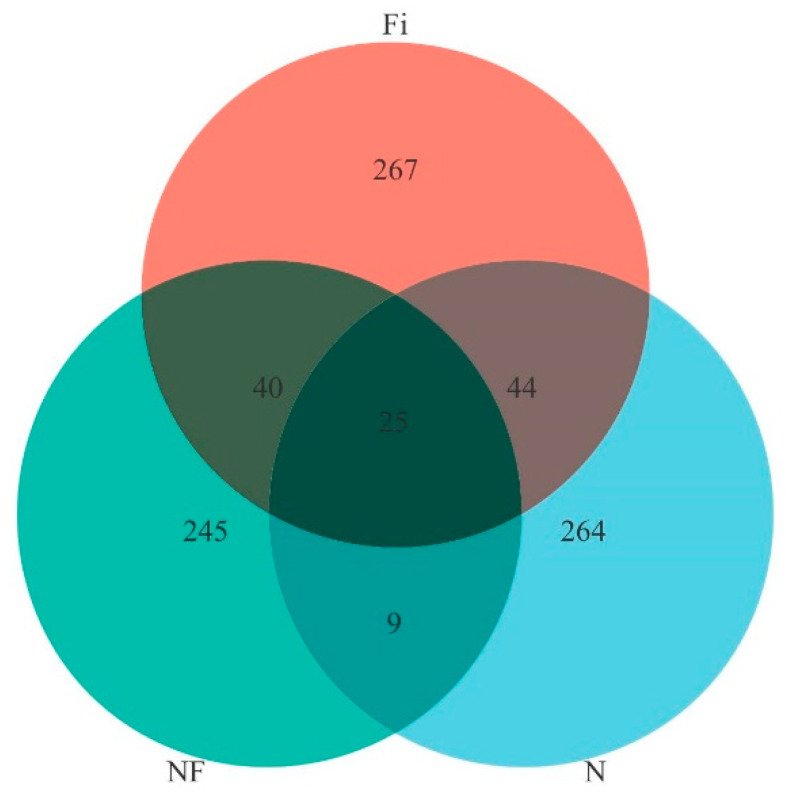
Venn diagram comparing the observed ASV of intestinal bacterial communities from shared and unique to each *P. japonicus* group.

Higher Shannon, Chao 1, and Simpson indexes occurred when the shrimp in group F was compared to the shrimp in groups N and NF, but there were no significant differences in these three groups (Table 5, *p* > 0.05). The analysis of unique or shared ASVs of live food (N), mixed food (NF), and formulated food (F) demonstrated that 25 ASVs were common to the three groups. The number of ASVs unique to the N, NF, and F groups was 289, 270, and 292, respectively (Figure 4).

**Figure 5:**
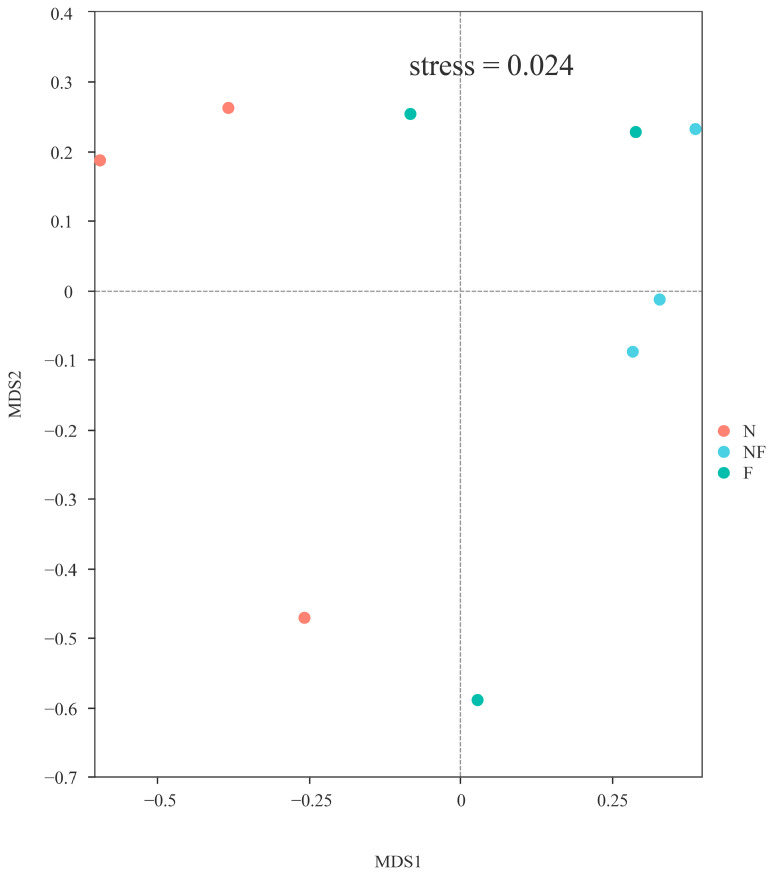
NMDS analyses based on weighted UniFrac distances between three groups, each point represented a sample, different color represented different groups.

Beta diversity was demonstrated by non-metric multi-dimensional scaling (NMDS); the stress value of 0.024 indicated a good representation of the dissimilarities in a two-dimensional space. Group NF was clustered more tightly than other groups, and the bacterial communities in groups F and NF were separated from group N (Figure 5).

**Figure 6:**
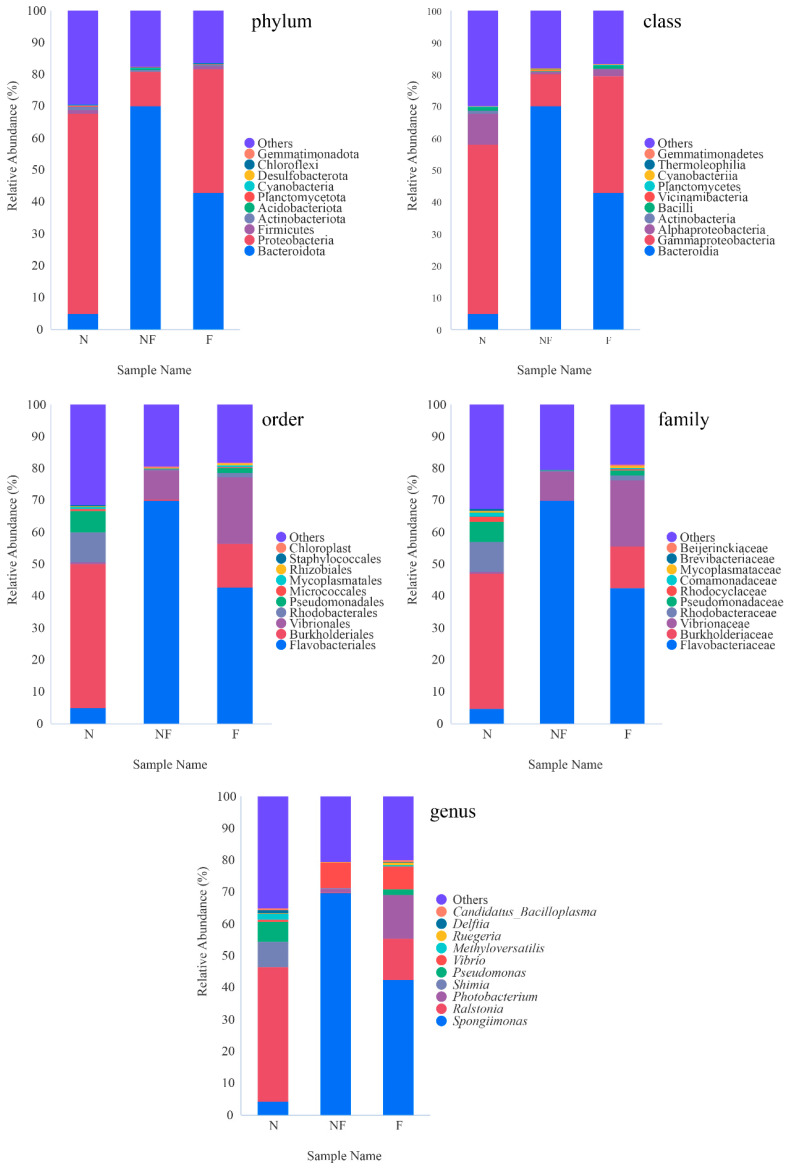
The relative taxa abundances of intestinal bacteria from phylum to genus levels in *P. japonicus*.

The relative taxa abundance of bacteria in the *P. japonicus* intestine is shown in Figure 6. The main phyla in the shrimp intestines among the three groups were Bacteroidota, Proteobacteria, Firmicutes, and Actinobacteriota. At the genus level, the prevalent microbial communities in the shrimp intestines consisted of *Spongiimonas*, *Ralstonia*, *Photobacterium*, *Shimia*, *Pseudomonas*, *Vibrio*, and *Methyloversatilis*. Compared with group N, the gut microbiota composition at different taxonomic levels was more consistent in both groups NF and F. Shrimp in group N showed lower relative abundances of the family Flavobacteriacea and its lineage (the phylum Bacteroides, the class Bacteroidia, the order Flavobacteriales) significantly (*p* < 0.05). Conversely, it exhibited higher relative abundances of the family Burkholderiaceae and its constituent genus *Ralstonia* (*p* < 0.05). Furthermore, compared to the N and NF groups, the F group exhibited higher relative abundance levels of the family Vibrionaceae and the genus *Photobacterium* (*p* < 0.05).

**Figure 7:**
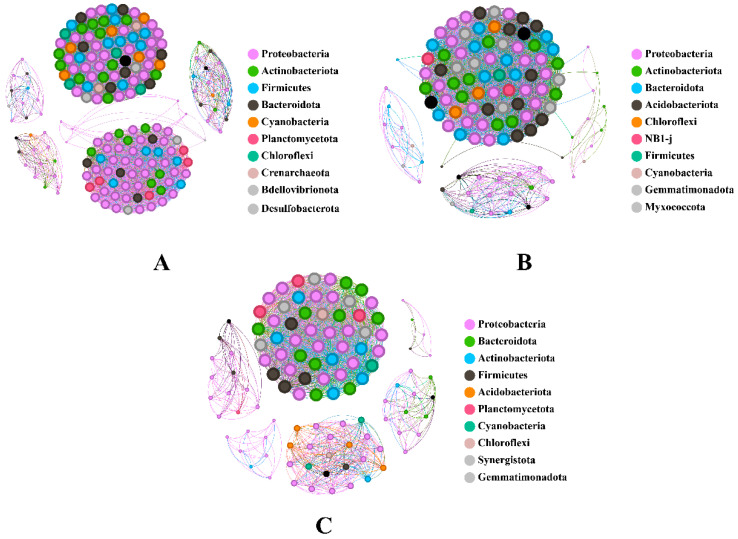
Co-occurrence network of shrimp intestine in different groups. (**A**): shrimp in group N, (**B**): shrimp in group NF, (**C**): shrimp in group F.

Shrimp in group N exhibited the largest and most complex network, with 181 nodes and 4755 edges (Figure 7A). In contrast, group NF had 113 nodes and 2897 edges (Figure 7B), and group F only had 111 nodes and 1727 edges (Figure 7C). Positive interactions dominated in all groups, with group N having the highest at 4662, followed by group NF with 2862, and group F with 1669. Group N had a lower density (0.29) compared to group NF, which had the highest density (0.45), and group F had the lowest density of 0.28 (Table 6).

## 4. Discussion

### 4.1. Differences in Growth Performance and Molting under Different Dietary Conditions

The growth of aquaculture has escalated the demand for formulated dietary resources, yet these diets often fall short of meeting the growth requirements of *P. japonicus*. In China, *P. aibuhitensis* is commonly used as live food for shrimp, especially in broodstock aquaculture [38,39]. Notably, *P. aibuhitensis* is rich in essential amino acids, more palatable to aquatic organisms, and contains a diverse range of fatty acids, including both monounsaturated and polyunsaturated types [40]. Given that shrimp have a limited capacity to synthesize unsaturated fatty acids [41,42], *P. aibuhitensis* can provide these crucial nutrients. This study analyzed the nutritional composition of live feed (*P. aibuhitensis*) and formulated feed, comparing their effects on the growth of *P. japonicus* through individual-rearing. The findings reveal that *P. aibuhitensis* exhibits superior crude protein and lipid levels, along with a more beneficial composition of essential amino acids and unsaturated fatty acids compared to formulated feed, aligning with previous studies. Feeding with *P. aibuhitensis*, solely or in a 1:1 mix with pellet diets, significantly enhanced *P. japonicus*’s growth performance and feed utilization. Proteins and lipids are vital for shrimp growth; *P. aibuhitensis* is rich in phospholipids and omega-3 fatty acids and acts as a feeding stimulant [43]. The proper composition of essential amino acids is also a key factor in promoting shrimp growth; *P. aibuhitensis* are good nutrition resources as live feed for shrimp to obtain better growth and maturation performance since they contain high levels of unsaturated fatty acids, other phospholipids, hormones like prostaglandin, and protein, especially *n*-3 and *n*-6 PUFAs, which are believed to be essential for the growth and development of shrimps [44,45]. These research findings are consistent with our results.

Notably, *P. japonicus* fed a mixed diet demonstrated a lower daily protein requirement and a higher protein efficiency rate compared to those fed exclusively on live food or a pellet diet. This discrepancy may arise from the shrimp’s varied nutritional needs and a more balanced protein-to-lipid ratio in the mixed diet, leading to enhanced diet utilization efficiency. Furthermore, an analysis of individual *P. japonicus* feeding and growth revealed instances of protein efficiency exceeding 100%. This phenomenon could be linked to the presence of beneficial nutrients in sandworms beyond proteins that contribute to shrimp growth. Prior research has highlighted the critical role of ω3-highly unsaturated fatty acids in promoting weight gain in Penaeid shrimp [46]. Consequently, the rich nutritional profile of sandworms creates an optimal growth environment for *P. japonicus*.

Furthermore, we employed an individual-rearing method to prevent mortality resulting from interactions between individuals, ensuring that observed fatalities were attributable to dietary factors. Results indicated that shrimp exclusively fed pellets experienced higher mortality due to unsuccessful molting attempts, adversely affecting growth and survival. Ecdysis is the process of intermittently enlarging crustacean body mass, which is crucial for shrimp growth [47]. There are many factors that affect shrimp molting, of which nutrients are one of the important nutritional factors; some studies have shown that crustaceans accumulate energy and important substances from ingested food, which has an important influence on the endocrine regulation of the molt cycle, in which sterols and long-chain unsaturated fatty acids play an important role. Kumar suggested that a high dietary cholesterol level is recommended due to shortened molting intervals in mud crab larvae [48], and dietary fatty acids, especially C18:2, C20:5, and C22:6, and other long-chain polyunsaturated fatty acids are essential for crustaceans and have a significant impact on growth, reproduction, and molting [49]. This study showcased that live food offers richer sterol and fatty acid content, facilitating successful molting and promoting the molting cycle in shrimp compared to a pellet diet. The addition of live food to pellet feed notably increased the molting survival rate, consistent with prior research.

### 4.2. Enzyme Activity and Antioxidant Capacity of Shrimp under Different Dietary Conditions

The hepatopancreas is a vital organ in shrimp, essential for nutrient assimilation and maintaining normal function [50]. Shrimp growth hinges on nutritional utilization, with the digestion and absorption of nutrients being contingent on the activities of digestive enzymes in the hepatopancreas, notably amylase, lipase, and Trypsin [51,52]. This study found that shrimp fed live food and mixed diets exhibited a superior growth performance compared to those on a pellet diet. Correspondingly, the digestive enzyme activities in the hepatopancreas mirrored this pattern. Amylase is crucial for nutritional assimilation in the hepatopancreas [53] and showed significantly higher activity in shrimp fed live food and mixed diets than in those on a pellet diet, aligning with the observed growth performance. Given *P. japonicus*’s high dietary protein requirement, Trypsin, the predominant proteolytic enzyme in the shrimp hepatopancreas [54], exhibited elevated activity with live food, enhancing digestion and thereby contributing to improved growth. Additionally, the highest lipase activity was observed in shrimp fed live food, possibly reflecting the fat composition of the sandworms and pellet diet. These results indicate that live food significantly enhances shrimp growth performance, and incorporating a small amount of live food into the pellet diet markedly improves growth compared to a pellet-only diet.

The endogenous antioxidant system, comprising enzymes and other molecules, is pivotal in eliminating reactive oxygen species and protecting cells from oxidative stress [55,56]. Antioxidant enzymes constitute the primary defense against reactive oxygen species, and they have been extensively identified in shrimp, including T-SOD, GSH, CAT, MDA, GSH-Px, and GPX [57]. In this study, shrimp fed live food showed increased hepatopancreatic levels of GSH and CAT. T-SOD and GSH-Px are functional antioxidant enzymes widely present in living organisms, playing a vital role in defending against damage. The results showed a significant increase in T-SOD levels in the hepatopancreas when shrimp were fed live food and mixed diets. Moreover, GSH-Px activity was notably higher in shrimp fed live food than those on a pellet diet, with mixed diets also enhancing GSH-Px activity. In recent years, sandworms have been rich in a variety of active substances and have been well documented, and an important function of these active substances is antioxidants [58,59]; thus, when being fed live food, shrimp may have acquired additional active substances that have boosted their antioxidant capacities. However, MDA levels in the hepatopancreas have not differed significantly across diets. Despite the absence of an adaptive immune system in shrimp, they possess a robust innate immune system for protection [60,61]. PO, AKP, and LZM play crucial roles in the immune defense of shrimp. AKP is involved in the transfer and metabolism of phosphoric acid, serving as a vital metabolic regulatory enzyme in organisms [51]. The results demonstrated that the AKP activity of shrimp fed live food and mixed diets was higher than in those fed a pellet diet. PO represents the terminal component of a complex cascade of enzymes that function in non-self-recognition and host defense in arthropods [60]; the current study indicated a significantly higher activity of PO in shrimp fed live food and mixed diets compared to shrimp fed a pellet diet. These results indicate that a pellet-only diet may not suffice for optimal shrimp health, and supplementing with live food can bolster their immunity and antioxidant capacity.

### 4.3. Effects of Different Dietary Structures on the Characteristics of Shrimp Intestinal Microbiota

Intestinal microbiota plays an important role in the health status of the host because it is closely linked to nutrient absorption and utilization, mucosal modification, and pathogen defense, which are necessary for the growth and health of the shrimp [62,63,64,65,66]. In aquaculture, the impact of different feed types on the intestinal microbiota of aquatic animals has been extensively studied [67,68,69]. Particularly, certain studies have examined the effects of live and formulated feeds on the intestinal microbial communities in aquatic species [70,71,72]. This study compared the differences in the intestinal microbial community composition of *P. japonicus* under live feed, formulated feed, and mixed feeding regimes. A total of 25 shared ASVs were detected in the intestinal microbiota of three *P. japonicus* groups, and the ASVs of the intestinal microbiota were higher in group F than those in groups N and NF. Furthermore, seen from the phylum to the genus, the sorts of microbiota in shrimp among the three groups shared a high similarity index, and the results revealed that the dominant bacteria in the intestinal tract of *P. japonicus* were mainly Bacteroidota, Proteobacteria, Firmicutes, and Actinobacteriota, which is consistent with the results of previous studies [55,73]. However, the relative abundances of intestinal microbiota varied with different treatments significantly. Compared to the other two groups, the shrimp in group N had a lower relative abundance of Bacteroidota. Previous studies have shown that higher ratios of Frimicutes to Bacteroidota improve nutrient transportation and digestion, which aligns with our observation of higher digestive enzyme activity in the shrimp from group N [64,74,75].

In healthy shrimp, both opportunistic pathogens and beneficial bacteria exist in the intestine [76]. Beneficial bacteria in the intestine usually improve the host’s health and promote the host’s nutrient absorption and immune response. This study identified the presence of several beneficial bacteria in *P. japonicus* intestines, including the yjr genus *Ruegeria* and *Shimia* and the order Rhodobacterales. The relative abundance of Rhodobacterales significantly increased in group N shrimps. The bacterial polyhydroxybutyrate (PHB) produced by Rhodobacterales might serve as an energy source for aquatic animals, which improves growth [64,77]. Similarly, live feed enhanced the relative abundance of *Shimia* in the shrimp intestines, which have an essential role in nutrient absorption, intestinal health, and improved growth in aquatic animals [78]. However, the shrimp intestine also harbors opportunistic pathogens present in the shrimp intestines, which are typically benign components of the normal intestinal microbiota; however, under certain conditions, these bacteria can become pathogenic [79]. This study found the presence of opportunistic pathogens such as the genus *Vibrio*, *Photobacterium*, the family Flavobacteriaceae, and Vibrionaceae in the gut of *P. japonicus*, aligning with previous research findings [80]. Compared to feeding live feed, feeding formulated feed significantly increased the relative abundance of *Vibrio* and *Photobacterium*. Shrimp fed with live food obtained a better growth performance and antioxidant capacity due to the higher nutrient content; some evidence suggests that retarded shrimp or shrimp with poor nutrition are more prone to pathogen invasion [65,81]. Thus, this may explain the increased abundance of opportunistic pathogens in *P. japonicus* fed formulated feed.

Microbial co-occurrence networks provide insights into the potentially complex interactions among species within a community [82,83,84]. In this study, the differences in network parameters across the conditions suggest that different diets can significantly influence microbial community structures and interaction patterns. The higher complexity and balanced interaction types in group N may indicate a more resilient microbial ecosystem capable of adapting to environmental changes. Conversely, after mixed feeding of live feed and formulated feed, the higher network density in group NF suggests a tightly knit community that might be more efficient in nutrient utilization but potentially less resilient to changes in environmental conditions. Group F, with its simpler network, may indicate that the gut is in a system under stress or nutrient limitations when only formulated food is fed, which is reflected by fewer interactions and lower connectivity.

### 4.4. Individual Variations in Growth and Feeding of Shrimp

Social interaction is widely recognized as a significant factor in individual growth variation among various species [85,86]. Typically, dominant fish exhibit superior growth performance and behaviors that afford greater resource access, often hindering the growth of subordinate fish. In populations where cannibalism occurs, subordinate individuals are more prone to being cannibalized, leading to increased growth variations within the group. Additionally, stocking density significantly influences individual growth variation [87,88,89]. In the present study, *P. japonicus* were individually housed, revealing that in the absence of social interaction and cannibalism, there is still notable inter-individual variation in growth and feeding performance. Moreover, there were substantial differences in growth, food intake, and food conversion efficiency among individuals correlated with their initial body weights (Figure 3C). Generally, smaller individuals displayed lower ingestion rates, growth rates, and food conversion efficiencies compared to larger ones [32]. Notably, individual monitoring revealed that some individuals in the F group matched the average growth and feeding efficiency observed in the NF and N groups. This indicates that certain individuals adapt well to formulated feed, an insight potentially obscured in group rearing. This approach facilitates the accurate selection of shrimp with better adaptability for further research. Similarly, selective breeding research on feeding efficiency traits using individual-rearing methods has also seen significant advancements [90,91,92].

Feeding significantly influences shrimp growth. This study identified considerable variations among individuals in feeding intake and feed conversion rates, which are both strongly linked to growth. Similar observations were made in Pacific white shrimp *Litopenaeus vannamei* reared individually, which exhibited substantial inter-individual differences in DFI, FER, and ADG; furthermore, high heritability estimates for these traits were reported [31]. Strong correlations between feed efficiency and growth rate have also been documented in terrestrial animals and fish, underscoring the general relevance of this relationship across species [93,94,95].

### 4.5. The Influence of Molting on Shrimp Feeding

Molting significantly influences the feeding behavior of shrimp. Unlike terrestrial animals and fish, shrimp demonstrate considerable day-to-day variability in feed intake throughout the production period. This study found that the shrimp’s DFI exhibits a cyclical pattern aligned with their molting cycle. It is well-documented that penaeid shrimp reduce feeding activity in the initial stages before and during molting [96,97]. Typically, the highest activity levels occur during the intermolt phase, decreasing as the shrimp approach the premolt phase, eventually reaching a state of inhibition; however, general activity remains high. Feeding and general activity levels plummet during molting. After molting, shrimp temporarily cease feeding, resuming normal activity as their exoskeleton hardens, aligning with our observations [98]. Despite considerable daily variations in individual feeding, the overall trend is consistent across individuals during each molting cycle. This consistency suggests new feeding strategies for shrimp culture. Research has indicated that adjusting the feed quantity based on molt status can optimize feed balance and protein efficiency in *L. vannamei*, underlining the importance of tailoring feeding to the molt cycle [99,100].

## 5. Conclusions

*P. japonicus* requires balanced nutrition for optimum growth. Despite the high protein content in commercial feed, it may not sufficiently fulfill all growth requirements. Our findings demonstrate that live food enhances shrimp growth, digestion, and immunity, and even a small addition of live food to the commercial diet significantly improves growth and immune function in *P. japonicus*. To our knowledge, this study is the first detailed investigation into how live and formulated feeds differently influence the intestinal microbiota composition in Kuruma shrimp (*P. japonicus*). The results indicate that feeding live feed increases the relative abundance of beneficial bacteria while decreasing opportunistic pathogens compared to formulated feed. Additionally, complementary feeding of live feed promoted a more tightly packed and complex network of intestinal flora in shrimp, enhancing their response to complex environmental changes.

Individual-rearing has proven effective in assessing the growth, feeding performance, and feed evaluation of *P. japonicus*. This approach allows for detailed growth and feeding data collection from individual shrimp, reducing the influence of non-dietary factors. Consequently, it offers a novel method for feed assessments and enables the exploration of individual growth and feeding variations. Additionally, the daily feed intake of *P. japonicus* shows consistent cyclical changes aligned with their molting cycle. These insights suggest that the nutritional profile of commercial feed is insufficient compared to live food, and the strategic inclusion of live food can significantly improve culture efficiency. The method of individual-rearing can effectively evaluate the growth and feeding performance of *P. japonicus* and conduct feed evaluation studies. Adjusting the diet based on the shrimp’s molting cycle could also optimize feed usage and enhance culture outcomes.

## Figures and Tables

**Table 1:** Nutritional composition of the live food (sandworms) and formulated pellet diet (% dry matter).

Ingredient (g/100 g)	Pellet Diet	Live Food
Proximate analysis		
Crude protein	46.73	59.00
Crude lipid	8.90	10.73
Moisture	10.16	80.10
Essential amino acids		
Threonine	1.52	1.74
Methionine	0.82	0.88
Valine	1.91	2.00
Isoleucine	1.57	1.79
Leucine	2.85	3.08
Phenylalanine	1.67	1.92
Histidine	0.99	0.97
Lysine	2.72	3.57
Arginine	2.18	2.90
Non-essential amino acids		
Aspartic acid	3.44	4.62
Serine	1.33	1.50
Glutamic acid	5.30	6.83
Glycine	2.33	2.54
Alanine	2.32	4.00
Cysteine	0.32	0.39
Tyrosine	1.04	1.40
Proline	1.87	2.88
TAA	34.16	43.01
EAA	16.22	18.86
NEAA	17.94	24.15
EAA/TAA	47.49	43.85
NEAA/TAA	52.51	56.15
Fatty acids (% fatty acids)		
C14:0	0.02	0.30
C16:0	1.89	1.64
C16:1n7	0.13	0.28
C18:0	0.57	0.44
C18:1n9	0.92	0.96
C18:2n6	1.27	1.22
C18:3n3	0.09	0.15
C20:4n6	0.22	0.07
C20:5n3 (DHA)	0.18	0.44
C22:1n9	0.02	0.02
C22:6n3 (EPA)	0.03	0.62
∑SFA	2.47	2.37
∑MUFA	1.05	1.23
∑PUFA	1.35	1.36
∑HUFA	0.42	1.13
DHA + EPA	0.21	1.06

TAA: sum of total amino acids. EAA: sum of essential amino acids NEAA: sum of non-essential amino acids. ∑SFA, saturated fatty acid: C16:0, C14:0, C18:0, C17:0. ∑MUFA, monounsaturated fatty acids: C16:1n-7, C18:1n-9, C18:1n-7, C20:1n-9. ∑PUFA, polyunsaturated fatty acid: C18:2n-6, C18:3n-3. ∑HUFA, highly unsaturated fatty acids: C20:4n-6, C20:5n-3, C22:5n-3, C22:6n-3.

**Table 2:** Differences in growth performance of shrimp under different feeding conditions after the 4 weeks feeding trial (n_N_ = 20, n_NF_ = 20, n_F_ = 17, respectively).

	N	NF	F
IBW/g	2.17 ± 0.14	2.11 ± 0.15	2.21 ± 0.24
FBW/g	4.20 ± 0.43 ^a^	4.20 ± 0.42 ^a^	3.18 ± 0.41 ^b^
WG/%	93.96 ± 14.35 ^a^	98.20 ± 9.86 ^a^	45.51 ± 18.43 ^b^
SGR %/day	2.27 ± 0.25 ^a^	2.35 ± 0.17 ^a^	1.26 ± 0.43 ^b^
FER	0.13 ± 0.02 ^a^	0.28 ± 0.06 ^b^	0.20 ± 0.05 ^c^
PER	0.98 ± 0.17 ^a^	1.06 ± 0.22 ^a^	0.44 ± 0.12 ^b^
FC/%	42.46 ± 2.37	41.70 ± 1.78	41.22 ± 1.51
SR/%	100.00 ^a^	100.00 ^a^	85.00 ^b^
3MR/%	100.00 ^a^	100.00 ^a^	57.89 ^b^

Values are means ± SD. ^a, b, c^ Mean values within a row with unlike superscript letters were significantly different (*p* < 0.05).

**Table 3:** Effects of different dietary groups on digestive enzyme of shrimp’s hepatopancreas (*n* = 3).

Digestive Enzyme	Groups		
	N	NF	F
ASM (U/g prot)	0.83 ± 0.08 ^a^	0.76 ± 0.02 ^a^	0.70 ± 0.05 ^b^
Trypsin (U/mg prot)	117.63 ± 46.13 ^a^	85.52 ± 10.01 ^b^	78.79 ± 2.93 ^c^
Lipase (U/g prot)	88.96 ± 14.35 ^a^	83.20 ± 9.86 ^a^	60.18 ± 8.43 ^b^

ASM, α-amylase. Values are means ± SD, ^a, b, c^ Mean values within a row with unlike superscript letters were significantly different (*p* < 0.05).

**Table 4:** Effects of different dietary group on immunity enzyme and antioxidant capacity of shrimp’s hepatopancreas (*n* = 3).

Immunity Enzyme	Groups		
	N	NF	F
AKP (U/g prot)	0.31 ± 0.09 ^a^	0.37 ± 0.14 ^a^	0.20 ± 0.07 ^b^
PO (ng/mL)	17.35 ± 1.27 ^a^	17.09 ± 1.16 ^a^	14.45 ± 0.85 ^b^
LZM (U/mg prot)	19.02 ± 3.32	18.37 ± 1.26	18.36 ± 8.46
T-SOD (U/mg prot)	188.95 ± 49.07 ^a^	121.87 ± 12.56 ^b^	87.31 ± 2.52 ^c^
CAT (U/mg prot)	13.33 ± 13.87 ^a^	8.15 ± 7.76 ^b^	8.78 ± 9.35 ^b^
GSH-Px (U/mg prot)	244.48 ± 51.56 ^a^	143.47 ± 48.36 ^b^	119.81 ± 33.43 ^c^
GSH (U/mg prot)	405.59 ± 153.84 ^a^	501.94 ± 112.20 ^a^	155.74 ± 39.43 ^b^
MDA (U/mg prot)	16.63 ± 7.96	23.07 ± 9.97	14.86 ± 7.81

AKP, alkaline phosphatase; PO, phenoloxidase; LZM, lysozyme; T-SOD, total superoxide dismutase; CAT, catalase; GSH-Px, glutathione Peroxidase; GSH, reduced glutathione; MDA, malondialdehyde. Values are means ± SD, ^a, b, c^ Mean values within a row with unlike superscript letters were significantly different (*p* < 0.05).

**Table 5:** Effects of different diets on the diversity of intestinal microbiota in *P. japonicus* (*n* = 3).

Items (%)	N	NF	F
ASV Number	110–185	38–220	44–192
Shannon	1.97 ± 0.82	1.74 ± 1.03	2.07 ± 0.93
Chao1	136.25 ± 40.95	121.72 ± 92.74	138.98 ± 84.50
Simpson	0.48 ± 0.22	0.47 ± 0.27	0.55 ± 0.24

**Table 6:** Comparison of topological parameters of co-occurrence networks (*n* = 3).

Items	N	NF	F
Number of nodes	181	113	111
Number of edges	4755	2897	1727
Positive correlation number	4662	2862	1669
Negative correlation number	93	35	58
Average degree	52.54	51.27	31.11
Network density	0.29	0.45	0.28

## Data Availability

The data presented in this study are available on request from the corresponding author.

## References

[1] Shinn A., Pratoomyot J., Griffiths D., Trong T., Vu N.T., Jiravanichpaisal P., Briggs M. (2018). Asian shrimp production and the economic costs of disease. Asian Fish. Sci..

[2] Cheng W., Zhang H., Wang P., Wei Y., Chen C., Hou Y., Deng X., Li S., Sun S., Cai Q. (2022). The Multiple Influences of Natural Farming Environment on the Cultured Population Behavior of Kuruma Prawn, *Penaeus japonicus*. Animals.

[3] FAO (2024). The State of World Fisheries and Aquaculture 2024. Blue Transformation in Action.

[4] Zheng J., Li L., Dong H., Mao Y., Su Y., Wang J. (2018). Molecular cloning of heat shock protein 60 from *Marsupenaeus japonicus* and its expression profiles at early developmental stages and response to heat stress. Aquac. Res..

[5] Zhang H., Cheng W., Zheng L., Wang P., Liu Q., Li Z., Li T., Wei Y., Mao Y., Yu X. (2020). Identification of a group D anti-lipopolysaccharide factor (ALF) from kuruma prawn (*Marsupenaeus japonicus*) with antibacterial activity against *Vibrio parahaemolyticus*. Fish Shellfish. Immunol..

[6] Hamasaki K., Kitada S. (2006). A review of kuruma prawn *Penaeus japonicus* stock enhancement in Japan. Fish. Res..

[7] Alam M.S., Teshima S.-i., Ishikawa M., Hasegawa D., Koshio S. (2004). Dietary arginine requirement of juvenile kuruma shrimp *Marsupenaeus japonicus* (Bate). Aquac. Res..

[8] Bulbul M., Kader M.A., Asaduzzaman M., Ambak M.A., Chowdhury A.J.K., Hossain M.S., Ishikawa M., Koshio S. (2016). Can canola meal and soybean meal be used as major dietary protein sources for kuruma shrimp, *Marsupenaeus japonicus*?. Aquaculture.

[9] Bulbul M., Kader M.A., Koshio S., Ishikawa M., Yokoyama S. (2014). Effect of replacing fishmeal with canola meal on growth and nutrient utilization in kuruma shrimp *Marsupenaeus japonicus* (Bate). Aquac. Res..

[10] Gamboa-Delgado J. (2022). Isotopic techniques in aquaculture nutrition: State of the art and future perspectives. Rev. Aquac..

[11] Deshimaru O., Shigeno K. (1972). Introduction to the artificial diet for prawn *Penaeus japonicus*. Aquaculture.

[12] Deng J., Ma S., Niu H., Dong S., Su Y. (2007). An experiment of shrimp (*Fenneropenaeus chinensis*) culture by inputting polychaetes (*Perinereis aibuhitensis*). Trans. Oceanol. Limnol..

[13] Liu S., Liu Y., Yang H., You K., Chen M., Yu L. (2006). Effects of *Perinereis aibuhitensis* and *Eisenia foetida* on growth and immune parameters of the shrimp *Litopenaeus vannamei*. J. Fish. Sci. China.

[14] Shigueno K. (1972). Problems on Prawn Culture in Japan.

[15] Kanazawa A., Shimaya M., Kawasaki M., Kashiwada K.-i. (1970). Nutritional requirements of prawn. 1. Feeding on artificial diet. Bull. Jpn. Soc. Sci. Fish..

[16] Deshimaru O., Kuroki K. (1974). Studies on a purified diet for prawn. 1. Basal composition of diet. Bull. Jpn. Soc. Sci. Fish..

[17] Alam M.S., Teshima S., Koshio S., Ishikawa M. (2004). Effects of supplementation of coated crystalline amino acids on growth performance and body composition of juvenile kuruma shrimp *Marsupenaeus japonicus*. Aquac. Nutr..

[18] Wang W., Ishikawa M., Koshio S., Yokoyama S., Hossain M.S., Moss A.S. (2018). Effects of dietary astaxanthin supplementation on juvenile kuruma shrimp, *Marsupenaeus japonicus*. Aquaculture.

[19] Bulbul M., Kader M.A., Ambak M.A., Hossain M.S., Ishikawa M., Koshio S. (2015). Effects of crystalline amino acids, phytase and fish soluble supplements in improving nutritive values of high plant protein based diets for kuruma shrimp, *Marsupenaeus japonicus*. Aquaculture.

[20] Bulbul M., Koshio S., Ishikawa M., Yokoyama S., Abdul Kader M. (2015). Growth performance of juvenile kuruma shrimp, *Marsupenaeus japonicus* (Bate) fed diets replacing fishmeal with soybean meal. Aquac. Res..

[21] Oswald A.T., Ishikawa M., Koshio S., Yokoyama S., Moss A.S., Serge D. (2019). Nutritional evaluation of *Nannochloropsis* powder and lipid as alternative to fish oil for kuruma shrimp, *Marsupenaeus japonicus*. Aquaculture.

[22] Le Vay L., Rodriguez A., Kamarudin M., Jones D. (1993). Influence of live and artificial diets on tissue composition and trypsin activity in *Penaeus japonicus* larvae. Aquaculture.

[23] Jones D., Kurmaly K., Arshard A. (1987). Penaeid shrimp hatchery trials using microencapsulated diets. Aquaculture.

[24] Hewitt D., Duncan P.F. (2001). Effect of high water temperature on the survival, moulting and food consumption of *Penaeus (Marsupenaeus) japonicus* (Bate, 1888). Aquac. Res..

[25] Rahman S.H.A. (1996). Evaluation of various diets for the optimum growth and survival of larvae of the penaeid prawn *Penaeus japonicus* Bate. Aquac. Nutr..

[26] Nguyen B.T., Koshio S., Sakiyama K., Ishikawa M., Yokoyama S., Kader M.A. (2012). Effects of polychaete extracts on reproductive performance of kuruma shrimp, *Marsupenaeus japonicus* Bate.–Part II. Ovarian maturation and tissue lipid compositions. Aquaculture.

[27] Dai P., Kong J., Meng X., Luo K., Lu X., Chen B., Cao B., Luan S. (2019). Genotype by environment interaction for feed efficiency trait of the juvenile Pacific white shrimp *Litopenaeus vannamei* held in individuals vs. in groups. Aquaculture.

[28] Wu J., Namikoshi A., Nishizawa T., Mushiake K., Teruya K., Muroga K. (2001). Effects of shrimp density on transmission of penaeid acute viremia in Penaeus japonicus by cannibalism and the waterborne route. Dis. Aquat. Org..

[29] Schaefer F.J., Flues S., Meyer S., Peck M.A. (2017). Inter- and intra-individual variability in growth and food consumption in pikeperch, *Sander lucioperca* L., larvae revealed by individual rearing. Aquac. Res..

[30] Yang Z., Wei B., Liu Q., Cheng Y., Zhou J. (2018). Individual growth pattern of juvenile stages of the Chinese mitten crab (*Eriocheir sinensis*) reared under laboratory conditions. Aquac. Int..

[31] Dai P., Luan S., Lu X., Luo K., Meng X., Cao B., Kong J. (2017). Genetic assessment of residual feed intake as a feed efficiency trait in the Pacific white shrimp *Litopenaeus vannamei*. Genet. Sel. Evol..

[32] Liang M., Dong S., Gao Q., Wang F., Tian X. (2010). Individual variation in growth in sea cucumber *Apostichopus japonicus* (Selenck) housed individually. J. Ocean. Univ. China.

[33] Dai P., Luan S., Lu X., Luo K., Cao B., Meng X., Kong J. (2017). Genetic evaluation of feed efficiency in the breeding population of *Fenneropenaeus chinensis* “Huanghai No. 2” using phenotypic, pedigree and genomic information. Aquac. Int..

[34] AOAC (2005). Official Methods of Analysis of the Association of Analytical Chemists International.

[35] Bokulich N.A., Subramanian S., Faith J.J., Gevers D., Gordon J.I., Knight R., Mills D.A., Caporaso J.G. (2013). Quality-filtering vastly improves diversity estimates from Illumina amplicon sequencing. Nat. Methods.

[36] Edgar R.C., Haas B.J., Clemente J.C., Quince C., Knight R. (2011). UCHIME improves sensitivity and speed of chimera detection. Bioinformatics.

[37] Wang Y., Guo H., Gao X., Wang J. (2021). The intratumor microbiota signatures associate with subtype, tumor stage, and survival status of esophageal carcinoma. Front. Oncol..

[38] Naessens E., Lavens P., Gomez L., Browdy C., McGovern-Hopkins K., Spencer A., Kawahigashi D., Sorgeloos P. (1997). Maturation performance of *Penaeus vannamei* co-fed *Artemia* biomass preparations. Aquaculture.

[39] Yang D., Wang C., Kou N., Xing J., Li X., Zhao H., Luo M. (2022). Gonadal maturation in *Litopenaeus vannamei* fed on four different polychaetes. Aquac. Rep..

[40] Techaprempreecha S., Khongchareonporn N., Chaicharoenpong C., Aranyakananda P., Chunhabundit S., Petsom A. (2011). Nutritional composition of farmed and wild sandworms, Perinereis nuntia. Anim. Feed. Sci. Technol..

[41] Clarke A. (1982). Lipid Synthesis and Reproduction in the Polar Shrimp *Chorismus antarcticus*. Mar. Ecol. Prog. Ser..

[42] Medina A., Vila Y., Mourente G., Rodríguez A. (1996). A comparative study of the ovarian development in wild and pond-reared shrimp, *Penaeus kerathurus* (Forskål, 1775). Aquaculture.

[43] Shan H., Zhao X., Zhou Y., Wang T., Ma S. (2019). Effects of freeze-dried powder of the Antarctic krill *Euphausia superba* on the growth performance, molting and fatty acid composition of the Pacific white shrimp *Litopenaeus vannamei*. Aquac. Res..

[44] Wouters R., Lavens P., Nieto J., Sorgeloos P. (2001). Penaeid shrimp broodstock nutrition: An updated review on research and development. Aquaculture.

[45] Meunpol O., Iam-Pai S., Suthikrai W., Piyatiratitivorakul S.J.A. (2007). Identification of progesterone and 17α-hydroxyprogesterone in polychaetes (*Perinereis* sp.) and the effects of hormone extracts on penaeid oocyte development in vitro. Aquaculture.

[46] Kanazawa A. Nutrition of penaeid prawns and shrimps. Proceedings of the First International Conference on the Culture of Penaeid Prawns/Shrimps.

[47] Kobayashi S. (2012). Molting growth patterns of the Japanese mitten crab *Eriocheir japonica* (de Haan) under laboratory-reared conditions. J. Crustac. Biol..

[48] Kumar V., Sinha A.K., Romano N., Allen K.M., Bowman B.A., Thompson K.R., Tidwell J.H. (2018). Metabolism and nutritive role of cholesterol in the growth, gonadal development, and reproduction of crustaceans. Rev. Fish. Sci. Aquac..

[49] Lemos D., Weissman D. (2020). Moulting in the grow-out of farmed shrimp: A review. Rev. Aquac..

[50] Wang W., Ishikawa M., Koshio S., Yokoyama S., Dawood M.A.O., Hossain M.S., Zaineldin A.I. (2019). Interactive effects of dietary astaxanthin and cholesterol on the growth, pigmentation, fatty acid analysis, immune response and stress resistance of kuruma shrimp (*Marsupenaeus japonicus*). Aquac. Nutr..

[51] Zhuang Y., Huang H., Liu X.L., Wang N.A., Zhong G.F. (2022). Effect of bovine lactoferricin on the growth performance, digestive capacity, immune responses and disease resistance in Pacific white shrimp, *Penaeus vannamei*. Fish Shellfish. Immunol..

[52] Wang Y., Abdullah, Zhang C., Li Y., Zhang H., Wang J., Feng F. (2020). Effects of dietary glycerol monolaurate on the growth performance, digestive enzymes, body composition and non-specific immune response of white shrimp (*Litopenaeus vannamei*). Aquac. Rep..

[53] Shao J., Liu M., Wang B., Jiang K., Wang M., Wang L. (2017). Evaluation of biofloc meal as an ingredient in diets for white shrimp *Litopenaeus vannamei* under practical conditions: Effect on growth performance, digestive enzymes and TOR signaling pathway. Aquaculture.

[54] Muhlia-Almazán A., Garcıa-Carreno F.L., Sanchez-Paz J.A., Yepiz-Plascencia G., Peregrino-Uriarte A.B. (2003). Effects of dietary protein on the activity and mRNA level of trypsin in the midgut gland of the white shrimp *Penaeus vannamei*. Comp. Biochem. Physiol. Part B Biochem. Mol. Biol..

[55] Zhang X., Li M., Tao X., Yang Y., Sun P., Jin M., Zhou Q., Jiao L. (2022). Effects of dietary montmorillonite supplementation on the growth performance, antioxidant capacity, intestinal barrier and microbiota composition in *Marsupenaeus japonicus*. Aquaculture.

[56] Parrilla-Taylor D.P., Zenteno-Savín T. (2011). Antioxidant enzyme activities in Pacific white shrimp (*Litopenaeus vannamei*) in response to environmental hypoxia and reoxygenation. Aquaculture.

[57] Wang D., Li F., Chi Y., Xiang J. (2012). Potential relationship among three antioxidant enzymes in eliminating hydrogen peroxide in penaeid shrimp. Cell Stress Chaperones.

[58] Deng Z., Wang S., Li Q., Ji X., Zhang L., Hong M. (2010). Purification and characterization of a novel fibrinolytic enzyme from the polychaete, *Neanthes japonica* (Iznka). Bioresour. Technol..

[59] Zhang W., Wang Z., Ganesan K., Yuan Y., Xu B. (2022). Antioxidant activities of aqueous extracts and protein hydrolysates from marine worm Hechong (*Tylorrhynchus heterochaeta*). Foods.

[60] Wang J., Zhang H., Yang Q., Tan B., Dong X., Chi S., Liu H., Zhang S. (2020). Effects of replacing soybean meal with cottonseed meal on growth, feed utilization and non-specific immune enzyme activities for juvenile white shrimp, *Litopenaeus vannamei*. Aquac. Rep..

[61] Lee S.Y., Söderhäll K. (2002). Early events in crustacean innate immunity. Fish Shellfish. Immunol..

[62] Soriano E.L., Ramírez D.T., Araujo D.R., Gómez-Gil B., Castro L.I., Sánchez C.G. (2018). Effect of temperature and dietary lipid proportion on gut microbiota in yellowtail kingfish *Seriola lalandi* juveniles. Aquaculture.

[63] Guo H., Huang L., Hu S., Chen C., Huang X., Liu W., Wang S., Zhu Y., Zhao Y., Zhang D. (2020). Effects of carbon/nitrogen ratio on growth, intestinal microbiota and metabolome of shrimp (*Litopenaeus vannamei*). Front. Microbiol..

[64] Zheng L., Xie S., Zhuang Z., Liu Y., Tian L., Niu J. (2021). Effects of yeast and yeast extract on growth performance, antioxidant ability and intestinal microbiota of juvenile Pacific white shrimp (*Litopenaeus vannamei*). Aquaculture.

[65] Xiong J., Dai W., Zhu J., Liu K., Dong C., Qiu Q. (2017). The underlying ecological processes of gut microbiota among cohabitating retarded, overgrown and normal shrimp. Microb. Ecol..

[66] Yan F.-j., Tian X.-l., Dong S.-l., Fang Z.-h., Yang G.J.A. (2014). Growth performance, immune response, and disease resistance against *Vibrio splendidus* infection in juvenile sea cucumber *Apostichopus japonicus* fed a supplementary diet of the potential probiotic *Paracoccus marcusii* DB11. Aquaculture.

[67] Bruni L., Pastorelli R., Viti C., Gasco L., Parisi G. (2018). Characterisation of the intestinal microbial communities of rainbow trout (*Oncorhynchus mykiss*) fed with *Hermetia illucens* (black soldier fly) partially defatted larva meal as partial dietary protein source. Aquaculture.

[68] Duan Y., Zhang Y., Dong H., Wang Y., Zhang J. (2017). Effect of the dietary probiotic *Clostridium butyricum* on growth, intestine antioxidant capacity and resistance to high temperature stress in kuruma shrimp *Marsupenaeus japonicus*. J. Therm. Biol..

[69] Wang H., Hu X., Zheng Y., Chen J., Tan B., Shi L., Zhang S. (2022). Effects of replacing fish meal with cottonseed protein concentrate on the growth, immune responses, digestive ability and intestinal microbial flora in *Litopenaeus vannamei*. Fish Shellfish. Immunol..

[70] Chen X., Sun C., Dong J., Li W., Tian Y., Hu J., Ye X. (2022). Comparative Analysis of the Gut Microbiota of Mandarin Fish (*Siniperca chuatsi*) Feeding on Compound Diets and Live Baits. Front. Genet..

[71] Chen X., Yi H., Liu S., Zhang Y., Su Y., Liu X., Bi S., Lai H., Zeng Z., Li G. (2021). Probiotics improve eating disorders in mandarin fish (*Siniperca chuatsi*) induced by a pellet feed diet via stimulating immunity and regulating gut microbiota. Microorganisms.

[72] Chen X., Yi H., Liu S., Zhang Y., Su Y., Liu X., Bi S., Lai H., Zeng Z., Li G. (2021). Promotion of pellet-feed feeding in mandarin fish (*Siniperca chuatsi*) by *Bdellovibrio bacteriovorus* is influenced by immune and intestinal flora. Aquaculture.

[73] Niu G.-J., Yan M., Li C., Lu P.-y., Yu Z., Wang J.-X. (2022). Infection with white spot syndrome virus affects the microbiota in the stomachs and intestines of kuruma shrimp. Sci. Total Environ..

[74] Zhang C., Zhang M., Pang X., Zhao Y., Wang L., Zhao L. (2012). Structural resilience of the gut microbiota in adult mice under high-fat dietary perturbations. ISME J..

[75] Murphy E., Cotter P., Healy S., Marques T.M., O'sullivan O., Fouhy F., Clarke S., O'toole P., Quigley E.M., Stanton C. (2010). Composition and energy harvesting capacity of the gut microbiota: Relationship to diet, obesity and time in mouse models. Gut.

[76] Li E., Xu C., Wang X., Wang S., Zhao Q., Zhang M., Qin J.G., Chen L. (2018). Gut microbiota and its modulation for healthy farming of Pacific white shrimp *Litopenaeus vannamei*. Rev. Fish. Sci. Aquac..

[77] Yamazaki Y., Meirelles P.M., Mino S., Suda W., Oshima K., Hattori M., Thompson F.L., Sakai Y., Sawabe T., Sawabe T. (2016). Individual *Apostichopus japonicus* fecal microbiome reveals a link with polyhydroxybutyrate producers in host growth gaps. Sci. Rep..

[78] Barreto-Curiel F., Ramirez-Puebla S.T., Ringø E., Escobar-Zepeda A., Godoy-Lozano E., Vazquez-Duhalt R., Sanchez-Flores A., Viana M.T. (2018). Effects of extruded aquafeed on growth performance and gut microbiome of juvenile *Totoaba macdonaldi*. Anim. Feed. Sci. Technol..

[79] Derome N., Gauthier J., Boutin S., Llewellyn M. (2016). Bacterial opportunistic pathogens of fish. The Rasputin Effect: When Commensals Symbionts Become Parasitic.

[80] Imaizumi K., Tinwongger S., Kondo H., Hirono I. (2021). Analysis of microbiota in the stomach and midgut of two penaeid shrimps during probiotic feeding. Sci. Rep..

[81] Xiong J., Zhu J., Dai W., Dong C., Qiu Q., Li C. (2017). Integrating gut microbiota immaturity and disease-discriminatory taxa to diagnose the initiation and severity of shrimp disease. Environ. Microbiol..

[82] Berry D., Widder S. (2014). Deciphering microbial interactions and detecting keystone species with co-occurrence networks. Front. Microbiol..

[83] Dai W., Zhang J., Tu Q., Deng Y., Qiu Q., Xiong J. (2017). Bacterioplankton assembly and interspecies interaction indicating increasing coastal eutrophication. Chemosphere.

[84] Banerjee S., Kirkby C.A., Schmutter D., Bissett A., Kirkegaard J.A., Richardson A.E. (2016). Network analysis reveals functional redundancy and keystone taxa amongst bacterial and fungal communities during organic matter decomposition in an arable soil. Soil Biol. Biochem..

[85] Martins C.I.M., Aanyu M., Schrama J.W., Verreth J.A.J. (2005). Size distribution in African catfish (*Clarias gariepinus*) affects feeding behaviour but not growth. Aquaculture.

[86] Alanärä A., Burns M.D., Metcalfe N.B. (2001). Intraspecific resource partitioning in brown trout: The temporal distribution of foraging is determined by social rank. J. Anim. Ecol..

[87] Galkanda-Arachchige H.S.C., Hussain A.S., Davis D.A. (2021). Improvement in laboratory research: Effects of stocking density, variation and sample size on outcomes of growth studies with shrimp. Aquac. Res..

[88] Rodríguez-Olague D., Ponce-Palafox J.T., Castillo-Vargasmachuca S.G., Arámbul-Muñoz E., de los Santos R.C., Esparza-Leal H.M. (2021). Effect of nursery system and stocking density to produce juveniles of whiteleg shrimp *Litopenaeus vannamei*. Aquac. Rep..

[89] Li Y., Li J., Wang Q. (2006). The effects of dissolved oxygen concentration and stocking density on growth and non-specific immunity factors in Chinese shrimp, *Fenneropenaeus chinensis*. Aquaculture.

[90] Besson M., Allal F., Chatain B., Vergnet A., Clota F., Vandeputte M. (2019). Combining individual phenotypes of feed intake with genomic data to improve feed efficiency in sea bass. Front. Genet..

[91] De Verdal H., Vandeputte M., Mekkawy W., Chatain B., Benzie J.A. (2018). Quantifying the genetic parameters of feed efficiency in juvenile Nile tilapia *Oreochromis niloticus*. BMC Genet..

[92] Yu W., Liu J., Yu F., Shen Y., Gong S., Lu Y., Peng W., Wang Y., Gan Y., Xiao Q. (2022). Heritability and genetic correlation for residual feed intake of Pacific abalone *Haliotis discus hannai*. Aquaculture.

[93] Kolstad K., Grisdale-Helland B., Gjerde B. (2004). Family differences in feed efficiency in Atlantic salmon (*Salmo salar*). Aquaculture.

[94] Santana M.H.A., Oliveira G.A., Gomes R.C., Silva S.L., Leme P.R., Stella T.R., Mattos E.C., Rossi P., Baldi F.S., Eler J.P. (2014). Genetic parameter estimates for feed efficiency and dry matter intake and their association with growth and carcass traits in Nellore cattle. Livest. Sci..

[95] Henryon M., Jokumsen A., Berg P., Lund I., Pedersen P.B., Olesen N.J., Slierendrecht W.J. (2002). Genetic variation for growth rate, feed conversion efficiency, and disease resistance exists within a farmed population of rainbow trout. Aquaculture.

[96] Chan S.-M., Rankin S.M., Keeley L.L. (1988). Characterization of the molt stages in *Penaeus vannamei*: Setogenesis and hemolymph levels of total protein, ecdysteroids, and glucose. Biol. Bull..

[97] Moss D.R., Moss S.M. (2006). Effects of Gender and Size on Feed Acquisition in the Pacific White Shrimp *Litopenaeus vannamei*. J. World Aquac. Soc..

[98] Bardera G., Usman N., Owen M., Pountney D., Sloman K.A., Alexander M.E. (2019). The importance of behaviour in improving the production of shrimp in aquaculture. Rev. Aquac..

[99] Molina C., Cadena E., Orellana F. (2000). Alimentación de Camarones en Relación a la Actividad Enzimática Como una Respuesta Natural al Ritmo Circadiano y Ciclo de Muda. https://www.dspace.espol.edu.ec/xmlui/bitstream/handle/123456789/8784/20030813.pdf.

[100] Vega-Villasante F., Nolasco-Soria H., Civera-Cerecedo R., González-Valdés R., Oliva-Suárez M. (2000). Alternativa para la alimentación del camarón en cultivo: El manejo de la muda. Avances en Nutrición Acuicola.

